# Algorithmic approaches to aid species' delimitation in multidimensional morphospace

**DOI:** 10.1186/1471-2148-10-175

**Published:** 2010-06-11

**Authors:** Thomas HG Ezard, Paul N Pearson, Andy Purvis

**Affiliations:** 1 Imperial College London, Silwood Park Campus, Ascot, Berkshire, SL5 7PY, UK; 2 School of Earth and Planetary Sciences, Cardiff University, Cardiff, CF10 3YE, UK

## Abstract

**Background:**

The species is a fundamental unit of biological pattern and process, but its delimitation has proven a ready source of argument and disagreement. Here, we discuss four key steps that utilize statistical thresholds to describe the morphological variability within a sample and hence assess whether there is evidence for one or multiple species. Once the initial set of biologically relevant traits on comparable individuals has been identified, there is no need for the investigator to hypothesise how specimens might be divided among groups, nor the traits on which groups might be separated.

**Results:**

Principal components are obtained using robust covariance estimates and retained only if they exceed threshold amounts of explanatory power, before model-based clustering is performed on the dimension-reduced space. We apply these steps in an attempt to resolve ongoing debates among taxonomists working on the extinct Eocene planktonic foraminifera *Turborotalia*, providing statistical evidence for two species shortly before the lineage's extinction near the Eocene/Oligocene boundary.

**Conclusion:**

By estimating variance robustly (samples containing incipient species are unlikely to be scaled optimally by means and standard deviations) and identifying thresholds relevant to a particular system rather than universal standards, the steps of the framework aim to optimize the chances of delineation without imposing pre-conceived patterns onto estimates of species limits.

## Background

Whilst the fundamental importance of the species remains paramount in systematic and evolutionary biology, debates on species concepts continue [1-3]. There is, however, a growing consensus that conceptualizing what species ought to be is different from delimiting them in practice [2,4,5]. Operationally, species delimitation pivots on the assumption that, once sexual dimorphism, ontogeny and other causes of group difference have been taken into account to leave directly comparable individuals for analysis (semaphoronts [6]), expression of genotypes or phenotypes [7] or both [8] should be more similar in two individuals of species X than between one individual of species X and one individual of species Y. Hence individuals within species are expected to cluster in genotypic or phenotypic space, with sparsely inhabited or empty space between them. The range of intra-species variation can surpass the range of inter-species variation [9] however, which is problematic for morphological species concepts which rely critically on the assumption that gaps between species do exist [1-3].

The development of molecular diagnostics to identify analogous gaps has been dramatic [10], yielding automated approaches to test specific evolutionary hypotheses and enabling rapid delimitation of previously-undescribed species [10,11] across taxa and biogeographical region [12]. Morphological data are nevertheless useful in delimiting species [13] and strong arguments exist to refine non-molecular approaches [14,15]. Morphological data are often the only sort available. Elsewhere, they can be integrated with genetic data to augment evidence for species delimitation [8] or provoke new hypotheses given a lack of congruence [16]. Morphological traits are the outcome of multi-locus variation, and thus constitute a more thorough reflection of variation among individuals than a particular, pre-determined section of the DNA sequence [14]. Furthermore, if divergent selection is the main driver of speciation, then adaptive (morphological) traits may provide the best insights into species' limits [17,18]. Whilst various approaches have been developed for delimiting species morphometrically using an *a priori *definition [19], development of hypothesis-driven morphometric techniques when there is no guide as to whether an individual in a genus belongs to species X or Y has been much slower than for equivalent molecular tests [10,20].

**Figure 1:**
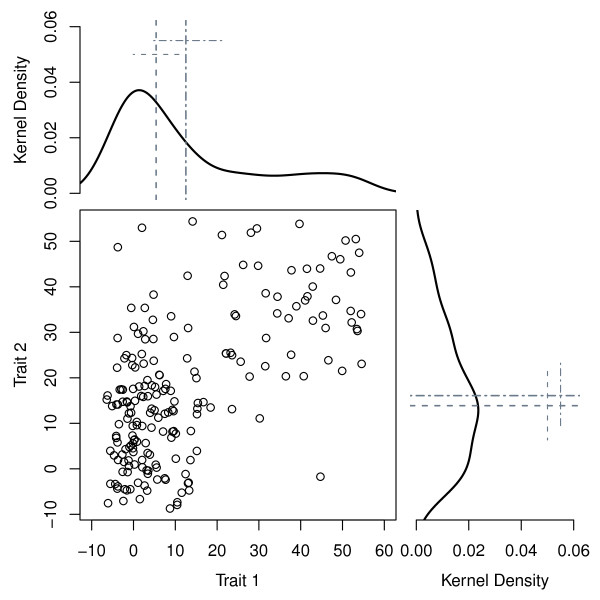
**How many clusters are there in a particular sample? **In this simulated instance (main panel), are there two, three or more? The problem is that adding additional clusters, by definition, explains more variation until each individual sits isolated in a cluster by itself [26]. If the cluster limits are robust, statistical methods should be able to identify them without resorting to *a priori *patterns. Cosine-smoothed kernel density plots [continuous analogues of histograms, [65]] highlight the difference induced by centering around the median (dot-dashed long lines) rather than the mean (dashed long lines) and scaling by the median absolute deviation rather than by standard deviations (the short, perpendicular lines are one median absolute deviation or standard deviation, as appropriate). In symmetric distributions, means and medians are similar, but median absolute deviations can still differ from standard deviations (right panel). If part of the population is an incipient species diverging from the majority, then medians and means also give different values (top panel).

To be suitable, any approach to this problem must be able to first characterize and then distinguish complex, multivariate organisms [19]. The mathematical translation of species delimitation is identification of well-separated clusters (Fig. 1): multiple species are inferred when two or more well-separated groups are a better way of describing a given sample than one group [21]. The problematic nature of non-discrete differentiation [9] has provoked the development of threshold models, which permit small numbers of supposedly distinct groups to show similar character expression: Wright [22] developed a threshold model to split continuous genotypic distributions into discrete character states; Simpson et al. [23] claimed that a coefficient of variation over 10 in a morphological trait indicated more than one taxon in a sample; Wiens & Servedio [24] used a 5% polymorphism cut-off to account for the rarity of characters being genuinely fixed; and Templeton [25] used a 5% homoplasy cut-off to partition data into independent haplotype networks. The use of threshold levels remains the crux of numerical taxonomy [7], but the idea that the appropriate threshold for species' delimitation is consistent across all questions and groups is problematic.

Any threshold measure effectively aims to identify how much "noise" can be discarded. In structured data, the identification of additional clusters increases within-group cohesion until, in the limit, each individual sits proudly as an isolated cluster [26] and no noise is discarded. Hence the problem becomes finding the optimal number of clusters to describe the structure parsimoniously. The simplest structure where an additional cluster is not warranted can be identified sequentially [e.g., are 3 clusters better than 2? [26,27]] or simultaneously [e.g., are 3 clusters better than 1,2,4,5, and so on? [28,29]]. These clusters may vary in their size (i.e., number of included individuals), shape and orientation [30]. Quantifying how much noise to discard relies heavily on the traits used to characterize differences among individuals. Any method to calculate variability within and between species should aim to neglect redundant measurements, whilst retaining essential differences. Accurate identification of the dimension and orientation of any dimension-reduced space is therefore a key problem.

In high-dimensional data, there often exists a lower-dimensional space that describes the majority of the observed variation, i.e., a smaller "fundamental set of independent variables ... which determine the values of the original data" [[31], cited in [32]]. Principal Components Analysis (PCA) finds a dimension-reduced space using variable covariance to rotate the original data to a new orientation that emphasizes similarities and differences among variables. Use of covariance for rotation assumes a multivariate normal distribution, which is often violated in taxonomic studies where data consist of a mixture of ratio, ordinal, binary and continuous variables, i.e. mixed mode, and might contain multiple species distributed in clumps across space. Many workers seek to circumvent this problem by using the correlation matrix rather than the covariance matrix as the basis for the PCA [ch. 11 in [32]], but outliers can still skew the orientation of the rotated axes markedly [33,34]. An alternative is to estimate variance robustly, using, for example, the median absolute deviation to detect the most variable direction [35]. The approach has already been employed in environmetrics [36] but not, to our knowledge, until now in systematic biology where it is relevant because samples containing incipient species are unlikely to be scaled optimally by means and standard deviations (Fig. 1).

Our approach to species delimitation consists of four steps: (1) obtaining orthogonal axes with robust covariance estimators [37]; (2) reducing the dimensionality of the orthogonal axes to only those with significant explanatory power [38]; (3) identifying the optimal number, shape and orientation of groups within the rotated, dimension-reduced data [28]; and (4) performing model diagnostics to assess the impact of unusual or extreme individuals on the dimension-reduced space and cluster model [39]. At each step, we use heuristic thresholds to retain or remove additional complexity, thereby minimising the scope for subjective choices. The details of application likely vary from question to question, but we argue here that they are key steps when splitting continuous morphological variation into discrete species. We first introduce the case study that motivated development, before going on to discuss application in this contentious area.

### Case Study - *Turborotalia cerroazulensis *lineage

**Figure 2:**
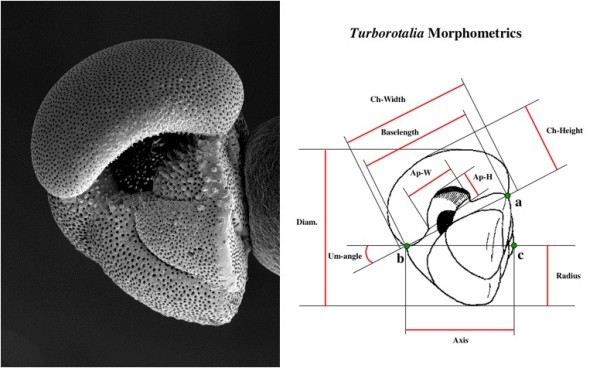
**The linear morphometric traits used (see Methods for a full list of incorporated traits), and an example *Turborotalia *specimen. **Of the morphotaxa discussed, this specimen most closely resembles *Turborotalia cerroazulensis*.

The *Turborotalia cerroazulensis *lineage (Fig. 2) constitute one of the most abundant and widely distributed groups of Eocene planktonic foraminifera [40]. Sine Bolli [41], Blow and Banner [42] and Toumarkine and Bolli [43], it has been widely appreciated that significant morphological change has occurred over geological time, which has made the group very useful for practical biostratigraphy [44]: earlier morphotypes tend to be more rounded in overall shape whereas later forms tend to be more angular and possess a distinct rim or keel around the periphery. Although a variety of biostratigraphically-important morphospecies have been described and the morphological differences between middle and upper Eocene forms are very obvious, it has been suggested that they are linked by populations comprising of overlapping morphospecies that intergrade temporally (*Turborotalia frontosa - T. possagnoensis - T. pomeroli - T. cerroazulensis - T. cocoaensis - T. cunialensis*) [43-46]. In this hypothesis, despite the group being split into six morphospecies, only a single morphological cluster is present at any one time. An alternative view [40] is that more than one species exists by the upper Eocene and hence genuine speciation and morphological divergence can be implied. The key problem is therefore to determine whether one or more morphological clusters are present at each time interval. Here, we restrict our investigation to two time slices, one from the older and one from the younger part of the succession (for more details, see Methods). Our aim is to demonstrate application to delimit species using the sorts of mixed mode data often found in 'traditional' taxonomic problems, especially in a palaeontological context when the morphological species concept is fundamental.

## Statistical Thresholds used to Delimit Species (STUDS)

### The Orientation of the Dimension-Reduced Space

The restrictive assumption of a multivariate normal distribution induced by use of covariance fails in many applied contexts, prompting refinement of the original PCA [see ch. 11 in [32]]. Using correlation matrices as an alternative does not always account for the potentially large effect that outliers can have on the orientation of the principal components, which aims to remove interdependence among components but can be biased by unusual observations [33,37]. As an illustration, Croux & Ruiz-Gazen [33] compared a PCA on the correlation matrix with a PCA using robust variance estimators, finding a correlation between the two "independent" components obtained from the correlation matrix argued to be a result of two outlying points. If scaled and centered data are not well-approximated by a multivariate normal distribution (multiple clusters within the data can also violate the normality assumption), rotated axes are more likely to pass through the centre of the multivariate data cloud when obtained using robust covariance estimators to identify the most variable traits using alternative metrics [35]. As the mixed mode data we analyze here are poorly described by the assumption of normality, each variable was centered on the median and scaled by percentile variability prior to rotation. Note that rotation is based upon the robust covariance estimates and pays no attention to the ultimate goal of finding cohesive clusters of comparable individuals - the implicit assumption is that the key distinctions among groups are present and identifiable in the traits used for analysis.

### Dimension Reduction

Retaining too few axes risks neglecting an influential one whereas retaining too many factors can deliver attention to relatively unimportant components (or, possibly, measurement error and inaccuracy [47]): either can generate bias, although the former is more serious [48]. Each retained variable should provide a significant improvement in explanatory power, whilst the improvement of each discarded variable should be minor: the threshold is located where the morphological trait information changes from being useful to irrelevant noise. Many stopping criteria have been proposed to determine where the threshold for dimension retention lies [see reviews in [38,47]] and no single measure outperforms all others [47]. Our case study contains multiple traits that are reasonably highly correlated. In such instances, adapting Horn's parallel analysis [49] to test whether the observed eigenvalues in the data are greater than the centiles of 10,000 Monte Carlo estimates is one of two most promising stopping criteria [[47], Additional file 1, online appendix 1 contains a comparison of multiple criteria and simulations documenting why dimension reduction is important in this context].

### Identification of well-separated clusters

The dimension-reduced sample can be split into groups using cluster analysis, the optimal delimitation of which has low intra- and high inter-group variability. K-means approaches [50,51] generate clusters with equal lengths in all dimensions (e.g., circles, spheres,....), which is not always representative of individuals in species clusters [30]. The flexibility in the shape and orientation of clusters can be incorporated using Gaussian mixture models and a Bayesian approach to estimate the support for particular arrangements of clusters using iterative Expectation-Maximization methods for maximum-likelihood [28]. The volume and shape can be equal or variable among axes to assess, for example, whether elliptical clusters fit better than round ones. The choice between competing models is made through the Bayesian Information Criterion (BIC, also known as the Schwarz Information Criterion), which is similar to the Akaike Information Criterion (AIC) and provides a compromise between explained variation and the number of parameters used [52]. The difference is that the penalty term per parameter for the BIC is log(n), where n is the number of observations, rather than 2 for the AIC. Consequently, BIC favours complex models more than AIC if n is at least 8, but simpler ones otherwise [52]. We present BIC values and also model weights, which sum to 1 and can be interpreted as the probability that a particular model provides the best description of the data structure among the set of candidate models [52].

### Model Diagnostics

Unusual or extreme individuals can exert great influence on the orientation of principal components [34,39]. The distances between individuals, when transformed to approach a chi-squared distribution, can be used to identify unusual observations at a desired level of significance [34]. Even if an individual is significantly unusual at a given level, there is still no guarantee that it unduly affects interpretation [39]; small groups of such points might rather be an under-sampled group that is diverging away from the main population.

### Implementation

All calculations were performed in R version 2.9.1 [53] and used the pcaPP [54], mvoutlier [34,54] and mclust [28] packages. A self-contained example of our code is included in Additional file 1, online appendix 1.

## Results

### Rotated and dimension-reduced morphospaces

The random average under parallel analysis criterion suggested that two components should be retained in both samples, which represented just under 50% of the variation in the original data in the upper Eocene but around 64% in the middle Eocene (Table 1). While test expansion and chamber aspect ratio were a key trait in both samples (identified using the loadings onto the robust principal components), filled (a shape variable, see Methods for full details) was a much stronger predictor in the upper Eocene than earlier in the sequence (Table 2). Area, the principal size measurement, loaded relatively weakly onto these components. These measurements reflect in different ways the main qualitative axis of variation used by taxonomists in morphospecies discrimination, i.e., the relative roundness of the shell versus the more angular, compressed morphology. The overall parametric correlation between loadings in the two samples was low (r = -0.124), which suggests substantial differences between typical individuals in the two samples.

**Table 1:** Based on stopping criteria, two components were retained in both samples (see Additional file 1, online appendix 1 for justification of stopping criteria and dimension reduction).

Component	Middle Eocene		Upper Eocene	
	Eigenvalue	Cumulative Variance Explained	Eigenvalue	Cumulative Variance Explained
1	3.47	0.29	5.97	0.42
2	2.33	0.49	3.24	0.64

3	1.43	0.61	1.44	0.74
4	1.27	0.72	0.95	0.81
5	1.15	0.81	0.74	0.86
6	0.75	0.88	0.68	0.91
7	0.5	0.92	0.5	0.94
8	0.42	0.95	0.31	0.97
9	0.42	0.99	0.31	0.99
10	0.09	1	0.11	1

**Table 2:** The loadings onto the robust principal components; larger absolute values indicate more influential traits on the dimension-reduced space.

Trait	Middle Eocene		Upper Eocene	
	Component 1	Component 2	Component 1	Component 2
Area	0.111	-0.355	0.289	-0.364
Filled	-0.508	0.239	0.382	0.317
Chamber Aspect Ratio	-0.395	-0.438	-0.418	0.453
Chamber Inflation	-0.390	-0.396	-0.274	0.062
Aperture Aspect Ratio	-0.176	-0.026	-0.249	0.148
Test Height	-0.261	0.374	0.470	0.220
Test Expansion	-0.505	0.228	-0.112	0.469
Umbilical Angle	0.103	0.522	-0.361	-0.047
Chirality	0.043	-0.037	0.122	0.192
Chamber Number	0.233	-0.048	-0.284	-0.478

### Sample Structure

Clustering on the dimension-reduced morphospaces found strong evidence to reject the null hypothesis of homogeneous data in the upper Eocene sample, but not in the middle Eocene (Table 3).

**Table 3:** Bayesian Information Criterion values and, in brackets, model weights for the model-based clustering based on robust principal components for the two samples.

Distribution	Model	Middle Eocene				Upper Eocene			
		1	2	3	4	1	2	3	4
**Spherical**	**E--**	1574 (0.010)	1575.5 (0.004)						
**Spherical**	**V--**	1574 (0.010)							1649 (0.002)
**Diagonal**	**EE-**	1567 (0.227)							
**Diagonal**	**VE-**	1567 (0.227)							
**Diagonal**	**EV-**	1567 (0.227)							
**Diagonal**	**VV-**	1567 (0.227)							
**Ellipsoidal**	**EEE**	1573 (0.017)							
**Ellipsoidal**	**EEV**	1573 (0.017)					1639 (0.224)	1648 (0.003)	
**Ellipsoidal**	**VEV**	1573 (0.017)					1637 (0.611)	1641 (0.101)	
**Ellipsoidal**	**VVV**	1573 (0.017)					1642 (0.059)	1649 (0.002)	

Legend: Although models for up to 10 clusters were fitted, only the first three had at least one model with non-zero weight and we only show models for up to 4 clusters in the table. The model codes relate to, in order, the volume, shape and orientation of the clusters: E denotes equal and V variable such that VEV, e.g., is a model with clusters of equal shape but different volume and orientation. BIC values and weights are only given if model weights were non-zero.

**Figure 3:**
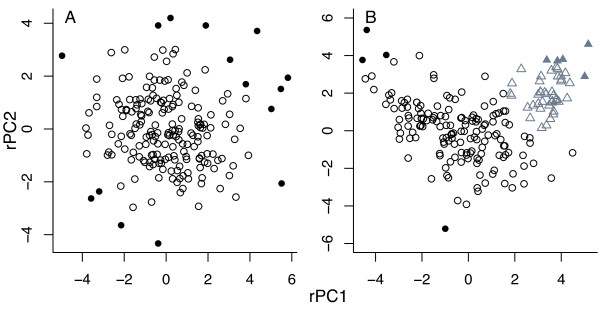
**Point clouds for the two samples show a homogeneous sample in the middle Eocene and a heterogeneous one in the upper Eocene. **The two clusters in the upper Eocene sample are shown in different symbols and colours. Significant outliers at the 5% level are denoted in solid symbols but were retained for all analyses, as conclusions were not altered qualitatively (see "Model Diagnostics").

**Figure 4:**
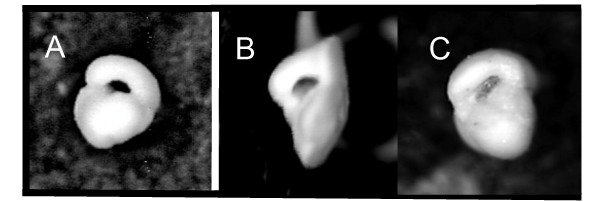
**Individuals at the centres of their clusters, identified as that with the minimum summed pairwise distance between it and all other individuals in their respective clusters. **Panel A is from the middle Eocene sample, with B and C being from the two upper Eocene clusters. These images have been cropped and are therefore no longer to scale; high-quality SEM images are available in [40].

The model weights in the earlier, middle Eocene sample suggested overwhelmingly that there was no evidence to reject the null hypothesis of one, mixed species (Fig. 3a): the model weight was 0.996 when summed across all one-cluster models (Table 3). The weights of all these one-cluster models were similar, meaning there is no clear inference of the shape of the three-dimensional point cloud. As previously described, the typical individual has a rounded overall shape (Fig. 4a).

**Figure 5:**
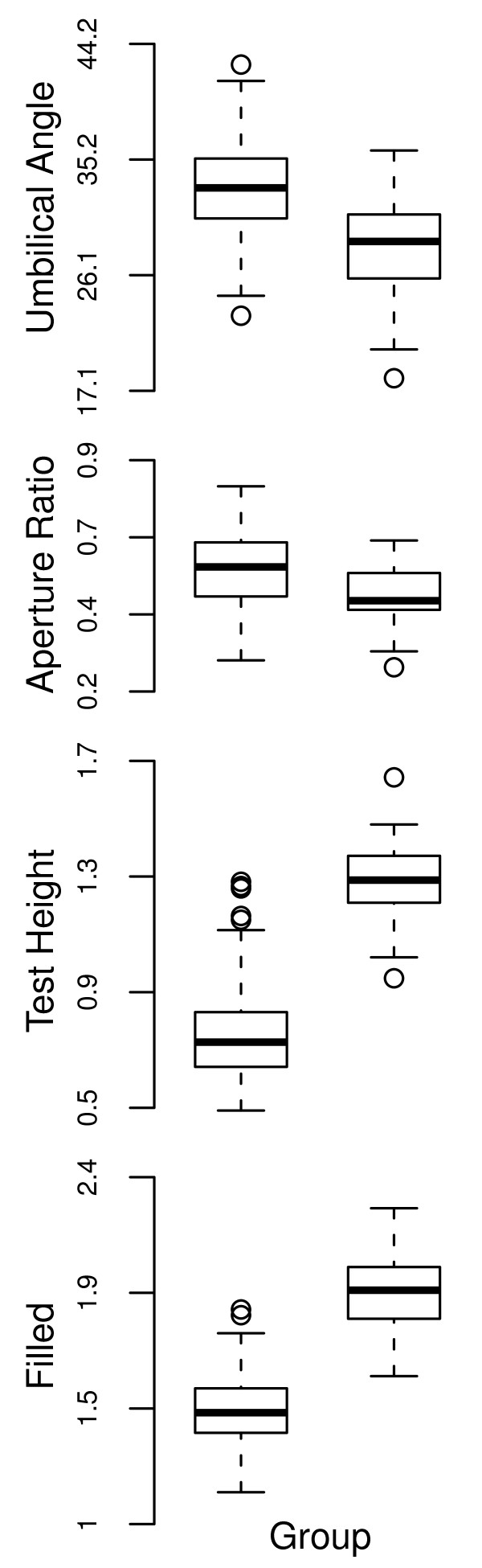
**The difference between the two clusters in the upper Eocene sample in four of the raw morphological traits was often clear**. The thick bar inside each box is the median, with the box extremities representing the inter-quartile range. The limits of each whisker are 1.5*(inter-quartile range), values beyond which are denoted by open circles

In contrast, the summed model weight of all one-cluster models for the upper Eocene sample was 0, with the remainder distributed among two- (0.894), three- (0.106) and four-cluster models (0.002). This is strong evidence to reject the null hypothesis in favour of the alternative hypothesis of more than one species in the genus at this time (Fig. 3b). As the three- and four-cluster model weights sum to 0.108 and these models are less parsimonious (they contain additional parameters compared to the two-cluster models), we do not discuss them further. The best model for the upper Eocene sample therefore favours two ellipsoidal clusters with equal shape (the model weight for this model alone was 0.611, Table 3) but of different abundance (155 and 45 individuals in each). Typical individuals in both are less rounded than earlier in the stratigraphic interval (Fig 4b-c), but key differences between the two upper Eocene groups are in test height, filled and aperture ratio (Fig. 5): smaller, less rounded individuals have a larger aperture ratio and less acute umbilical angle (the mean values are 1.313 μm vs. 1.984 μm, 0.849 vs. 1.362, 0.581 vs. 0.456 and 33.3° vs. 28.3°, respectively). In taxonomic terms the cluster containing more-rounded individuals corresponds more closely to the holotype of *Turborotalia cerroazulensis*, whereas the second corresponds to *T. cocoaensis *(see SEMs in [40] of these specimens).

## Discussion

Whilst automation at the expense of all else is undesirable [55], the use of taxonomically informative data should enable species' limits to be readily visualized [21]. We set out to test the null hypothesis that the species lineage of the Eocene planktonic foraminifer *Turborotalia *is homogeneous, i.e. one that contains an insensibly continuous series of intergrading populations [44] despite comprehensive description of multiple morphospecies regularly used in biostratigraphy [40]. In the middle Eocene sample, there was no statistical evidence to split the sample; this does not mean that multiple species are not present, just that there is no statistical evidence to delimit them. In the upper Eocene sample, the statistical evidence supported two species strongly (model weights ~0.9), whereas support for the existence of other numbers of species was very low. Delimitation is subtle enough to be difficult to determine by eye and requires investigation of large populations using multivariate data analysis. Crucially, we neither made *a priori *assumptions about the assignment of individuals to clusters nor employed prior knowledge of which traits determined those differences. Our use of robust variance estimators [37] is particularly appropriate for samples containing incipient species diverging in opposing directions (Fig. 1). Consequently, our approach is more general than this particular study: it can be used if a test is sought for the null hypothesis of multivariate homogeneity without specifying a particular alternative.

The increased adoption of geometric morphometrics - which utilizes distances among functionally-important characters referred to as landmarks - has been underpinned by a desire to quantify shape precisely across diverse questions, often to understand changes in shape independent of size [56] and can delimit previously-undetected species [57]. Size, however, often has a decisive role in diversification [58] and there is no guarantee that separating shape from it is biologically reasonable [59]. Our aim was to aid 'classical' morphometric treatments, where taxonomists have established decisive and informative functional traits. The steps discussed here serve to increase repeatability of taxonomic decisions, through the quantitative element that morphometric treatment adds to descriptions of sample variability. The connectivity of samples to be delimited can be achieved in many ways using, for example, Fourier analysis of distances between neighbours [60], neural networks [61] or, as here, by encompassing clusters of similar individuals using polygons [28]. The details of application may change on a case-by-case basis as there is rarely a one-size-fits-all recipe, but the steps of the framework have widespread utility in aiding species delimitation that might otherwise be obscured through mathematical conflation.

Distributions of biological traits are rarely described optimally when centered and scaled by means and standard deviations and rotated using covariance; such data are treated more appropriately using robust covariance estimates (Fig. 1). The use of robust covariance estimators to rotate raw data onto principal components is not widespread and yields a different dimension-reduced space from correlation- or covariance-based approaches [37]. There are alternatives to obtain appropriate scaling, notably principal co-ordinates analysis [62] but that method does not lend itself readily to threshold criteria, which are important to ensure parsimonious description of the untransformed traits in orthogonal space. Data can be, and often are, transformed prior to scaling, centering and rotation, but there is often no biologically meaningful transformation [63]. Whilst each variable could be transformed independently to obtain approximately symmetric, normally distributed distributions, doing so can inhibit interpretation. An advantage of robust approaches is that they de-emphasize extreme values: medians and median absolute deviations are less affected by long-tailed or asymmetric distributions than means and standard deviations. Both of these circumstances may arise during the early stages of divergence, when a population is splitting into incipient species. Inadequate scaling can skew the orientation of the principal components [35], hence obscuring potentially critical differences. The notion of being 'well-separated' applies to extreme data points as well as distinct clusters, meaning that identification of genuine outliers can be problematic. In the case studies we present here, we have no biological or methodological reason to remove the outliers, which might correspond to one or more undersampled or hidden groups. Without *a priori *grounds to eliminate them, results are reported here from analyses that retained these data and we note that the use of robust variance estimates means that manual removal is less likely to alter conclusions than standard, covariance-based approaches.

If species are argued to be morphologically distinct [1-3], then threshold-stopping criteria can be used to aid delimitation in morphospace. The failure to use prior knowledge of which individuals belong to which species and which traits are the key distinctions between species does not maximise power, but is based upon the argument that rejecting the null hypothesis (homogeneous data) in a contemporaneous sample implies two or more species. Analogous approaches have been applied to the problem of species' delimitation from alternative perspectives. Pons et al. [11] used changes in per-lineage branching rate to cluster tips on a phylogenetic tree, with the threshold between intra- and inter-species variation being the point where branching rates underwent a striking increase. Clusters in contemporaneous samples of comparable individuals are argued to be putative species [1-3]. The approach we present provides a useful analogue to methods that cluster tips on a phylogeny [11], from a bottom-up (samples into multiple clusters) rather than top-down (merging phylogenetic and phylogeographic methods) perspective for estimating sample structure in, amongst others, genotypic or phenotypic [7] or geographical [64] space. Application is likely to be particularly appropriate for species delimitation questions in the fossil record [57], or to test for congruence between morphological and genetic differentiation without resorting to using the other evidence as a prior hypothesis [8].

## Conclusion

Taxonomic decisions should ideally be taken by aligning state-of-the-art statistics with taxonomic expertise. Any technique to delimit species pivots on its ability to quantify the heterogeneity contained within species: "even an improved taxonomy still imposes structure on macroevolutionary investigation" [pp. 371, [60]] and the success of any statistical approach pivots on the use of biologically relevant data. The details of application will differ from question to question and from group to group, but the four steps discussed here decrease the potential for subjective decisions in species delimitation once biologically relevant traits have been identified. By scaling and centering appropriately, estimating variance robustly and identifying the thresholds that are relevant to the particular question and data set rather than a universal guide, the objective is to minimize the extent to which pre-conceived pattern is forced onto data.

## Methods

Raw data were obtained from Hole 865B of the Ocean Drilling Program (equatorial mid-Pacific Ocean). The entire data set consists of 51 time slices through a stratigraphic interval spanning around 13 million years. We focus on two samples here: one middle Eocene and one upper Eocene; a future contribution will analyze many more time slices and discuss the pattern of evolution in detail. Samples of 10 cm^3 ^were taken from the sediment and washed over a 63-micron sieve to remove fine particles (mainly coccoliths). The sieved residue is >99% planktonic foraminifer shells. All specimens of the *Turborotalia cerroazulensis *group of morphospecies were identified by eye using the taxonomic criteria of Pearson et al. [40] from multispecies assemblages and picked without further reference to species designation. Most other groups of foraminifera are easily distinguished, although rejection of specimens belonging to *T. altispiroides *and *T. ampliapertura *required a greater degree of expert discrimination [40]. The first 200 specimens encountered were manually separated and mounted on cardboard slides in a standard orientation (edge on, aperture facing). For each specimen, fine adjustments were made using a universal stage to achieve as consistent a standard orientation as possible. The choice of orientation in side view and measurements were carefully designed to capture the greatest range of morphological variability in the group, including the characters that are used in qualitative discrimination of the morphospecies by working taxonomists [40].

Measurements were made from photographs of each individual using Image Pro+ (Image Software, UK). The following morphological traits were incorporated in analysis: area, 'filled' (the proportion of a circle of an individual's radius filled by that individual), final chamber inflation (chamber width scaled by length), final chamber and aperture aspect ratio (the height: width ratio of the final chamber and aperture, respectively), test height (axis/radius), test expansion (diameter/radius), umbilical angle, chamber number and chirality. See fig. 2 for more information.

## Authors' contributions

THGE developed the framework, generated simulations, analyzed the data and produced new figures; PNP conceived the study and co-ordinated data collection; AP was involved in framework development; AP & PNP supervised the work; THGE, PNP and AP wrote the text. All authors read and approved the final manuscript.

## Supplementary Material

Additional file 1 **Further Methodological Detail**. This appendix contains (1) a comparison of different threshold-stopping criteria for use in dimension reduction; (2) simulation study to illustrate the importance of dimension reduction in cases like this; and (3) self-contained R code to follow the framework as implemented here. Click here for file
