# Supplementary material for: Algorithmic approaches to aid species' delimitation in multidimensional morphospace
Source: BMC Evol Biol. 2010 Jun 11;10:175. doi: 10.1186/1471-2148-10-175 (PMC2898690; doi:10.1186/1471-2148-10-175)
Supplement: Additional file 1 — Further Methodological Detail. This appendix contains (1) a comparison of different threshold-stopping criteria for use in dimension reduction; (2) simulation study to illustrate the importance of dimension reduction in cases like this; and (3) self-contained R code to follow the framework as implemented here. [file 1471-2148-10-175-S1.PDF]

Algorithmic Approaches to Aid Species' Delimitation in Multidimensional Morphospace: Online Appendix 1

Thomas H.G. Ezard, Paul N. Pearson and Andy Purvis

This appendix contains:

1. a comparison of different threshold-stopping criteria for use in dimension reduction;
2. a simulation study to illustrate the importance of dimension reduction in cases such as this;
3. self-contained R code to follow the framework as we implemented it here;
4. References.

1 Comparing Different Threshold Criteria

The problem of where the threshold between useful information and irrelevant noise in principal components has attracted extended attention, with rules being proposed and amended for well over half a century [1-6]. Retaining too few axes risks neglecting an influential one whereas retaining too many factors can deliver attention to relatively unimportant components: either can generate bias, although the former is more serious [1]. Each retained variable should provide a significant improvement in explanatory power, whilst each discarded variable should only cause incremental improvement. The performance of multiple methods has been rigorously investigated elsewhere [2, 3], concluding that no

single measure consistently outperforms all others, but that consistency among measures is greatest when variables are highly correlated [3].

In our samples, the tendency for Kaiser-Guttman to retain more components than other criteria is borne out, while the similarity in performance of the broken-stick and random average under parallel analysis is also expected when variables are highly correlated [3]. Correcting parallel analysis using random averages alters the recommended number of components in this instance because a number of the traits employed are highly correlated [3].

Method	Middle Eocene	Upper Eocene
**Kaiser-Guttman [4, 5]**	4	3
**Broken Stick [6]**	2	2
**Parallel Analysis [7]**	5	3
**Random Average under Parallel Analysis [3, 7]**	2	2

2 The Importance of Dimension Reduction

We use simulation studies to illustrate the importance of dimension-reduction in situations such as the one presented here, and follow the suggestion made in [3] that simulation studies should approximate the

situation at hand. That is one of numerous weakly correlated traits, but also several highly correlated ones. We restrict ourselves to one example of these situations, using three traits for ease of exposition.

We simulate three traits for two groups of individuals using gamma distributions (a typical example is given overleaf). Trait 3 is however merely the first trait with noise sampled from a uniform distribution, i.e. these two traits are highly correlated with one another:

**Trait 1**		
0.466	**Trait 2**	
0.849	0.396	**Trait 3**

We deliberately choose overlapping distributions to illustrate the power of the model-based clustering approach [8, 9] to delimit groups; the second cluster is restricted to lower values of trait 1 but can still extend throughout the approximate range of trait 2. A randomly selected simulation from the 1000 replicates used is:

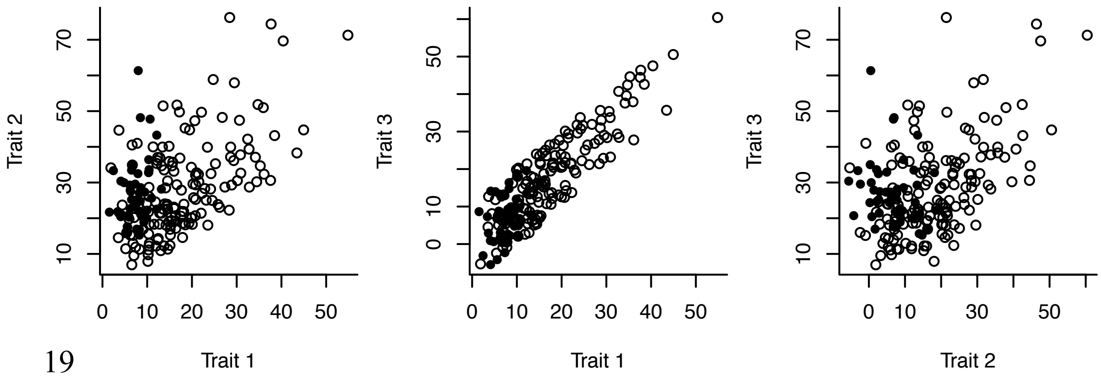

From 1000 replicates of the simulation, the median number of clusters when no dimension reduction was implemented was 3 (mean 3.061), whereas the median ***with*** dimension reduction was 2 (mean 2.414). In every case, there was support for dimension reduction using random average with parallel analysis [7] [3] and broken stick [6] methods: 1 component was retained in around 10% of cases with 2 components being retained in the remainder.

The Gamma distribution was chosen as it can be tweaked to approximate normal or skewed distributions by changing the size and/or shape parameters. The reason for the large skew in these distributions is that parameter values were chosen to approximate sections of populations diverging away from the majority (see Figure 1 in the main manuscript) and therefore extreme or unusual individuals were more likely. In the overwhelming majority of cases (>90%), the additional clusters have very low numbers of individuals.

An advantage of robust approaches is that they de-emphasise extreme values: medians and median absolute deviations are less affected by long-tailed or asymmetric distributions than means and standard deviations [10], hence obscuring potentially critical differences and restricting the ability to delimit species. The notion of being 'well-separated' applies to distinct clusters as well as extreme data points, meaning that identification of genuine outliers can be problematic. In these simulations, these individuals around the margins of the distributions may be genuine, statistical outliers or may reflect under-sampled or hidden groups.

3 Self-Contained R Code

```
      #you may need to install these packages first
library(pcaPP)# [11]
library(mclust)# [8]
library(mvoutlier)# [11, 12]
library(vegan)

      #generate some random data
t1 <- rgamma(150, 1.5, .1)
t2 <- rnorm(150, 10, 2)
tt <- matrix(c(t1, t2), ncol=2, byrow=FALSE)

      #fit the robust PCA using the GRID algorithm
m1 <- PCAgrid(tt, k=11, scale=mad, center=median, method="qn")
nk <- max(which(m1$sdev^2 > bstick(m1)))
nk    #this is the number of components to retain using broken stick

pp <- predict(m1)[,1:nk]
      #take the PCA predictions, i.e. rotated values, and cluster
m2 <- Mclust(pp)
m2    #this is the modelled structure

grps <- as.numeric(m2$classification)
out01 <- sign2(as.data.frame(pp), qcrit=.975)
out <- out01$wfinal01
      #this line creates a column of 0s and 1s whether an individual
      #..is a significant outlier at the 5% level of significance.

plot(tt, col=grps, pch=grps)
      #plot, with colour determined by model classification
```

4 References

1. Hayton JC, Allen DG, Scarpello V: **Factor Retention Decisions in Exploratory Factor Analysis: a Tutorial on Parallel Analysis.** *Organizational Research Methods* 2004, **7**(2):191-205.
2. Jackson DA: **Stopping rules in principal components analysis: a comparison of heuristical and statistical approaches**. *Ecology* 1993, **74**:2204-2214.
3. Peres-Neto PR, Jackson DA, Somers KM: **How many principal components? stopping rules for determining the number of non-trivial axes revisited**. *Comp Stat Data Anal* 2005, **49**:974-997.
4. Guttman L: **Some necessary conditions for common factor analysis**. *Psychometrika* 1954, **19**:149-162.
5. Kaiser HF: **The application of electronic computer to factor analysis.** *Educational and Psychological Measurement* 1960, **20**:141-151.
6. Frontier S: **Etude de la decroissance des valuers propers dans une analyze en composantes principales: comparison avec le modele de baton brise**. *Journal of Experimental Marine Biological Science* 1976, **25**:67-75.
7. Horn JL: **A rationale and a test for the number of factors in factor analysis**. *Psychometrika* 1965, **30**:179–185.
8. Fraley C, Raftery AE: **Model-based clustering, discriminant analysis, and density estimation**. *Journal of the American Statistical Association* 2002, **97**:611-631.
9. Oh M-S, Raftery AE: **Model-Based Clustering with Dissimilarities: A Bayesian Approach**. *Journal of Computational and Graphical Statistics* 2007, **16**(3):559-585.
10. Li G, Chen Z: **Projection-pursuit Approach to Robust Dispersion Matrices and Principal Components: Primary Theory and Monte Carlo**. *Journal of the American Statistical Association* 1985, **80**:759-766.
11. Filzmoser P, Garrett RG, Reimann C: **Multivariate outlier detection in exploration geochemistry**. *Computers & Geosciences* 2005, **31**:579-587.
12. Filzmoser P, Maronna R, Werner M: **Outlier identification in high dimensions.** *Comp Stat Data Anal* 2008, **52**:1694-1711.

The parallel analysis and random averages under parallel analysis criteria were implemented by adapting the paran package by Alexis Dinno. See doyenne.com/Software/index.shtml#paran and http://cran.r-project.org/web/packages/paran/index.html.

We do this by altering line 14 of the paran function to read:

```
mpp <- PCAgrid(xx, scale=mad, center=median
eigenvalues <- mpp$sdev^2
```

The previous line read:

```
eigen(cor(x), only.values = TRUE, EISPACK = FALSE)[[1]]
```
